# Supplementary material for: C3a Mediates Endothelial Barrier Disruption in Brain-Derived, but Not Retinal, Human Endothelial Cells
Source: Int J Mol Sci. 2024 Oct 19;25(20):11240. doi: 10.3390/ijms252011240 (PMC11508547; doi:10.3390/ijms252011240)
Supplement: Supplementary file 1 [file ijms-25-11240-s001.zip › ijms-3219137-supplementary.pdf]

C3a Mediates Endothelial Barrier Disruption in Brain-Derived,but Not Retinal, Human Endothelial Cells

Hannah NoraWolf †, Larissa Guempelein †, Juliane Schikora and Diana Pauly *

Department of Experimental Ophthalmology, University Marburg, 35043 Marburg, Germany
* Correspondence: Correspondence: diana.pauly@uni-marburg.de
† These authors contributed equally to this work.

SUPPLEMENTARY MATERIAL

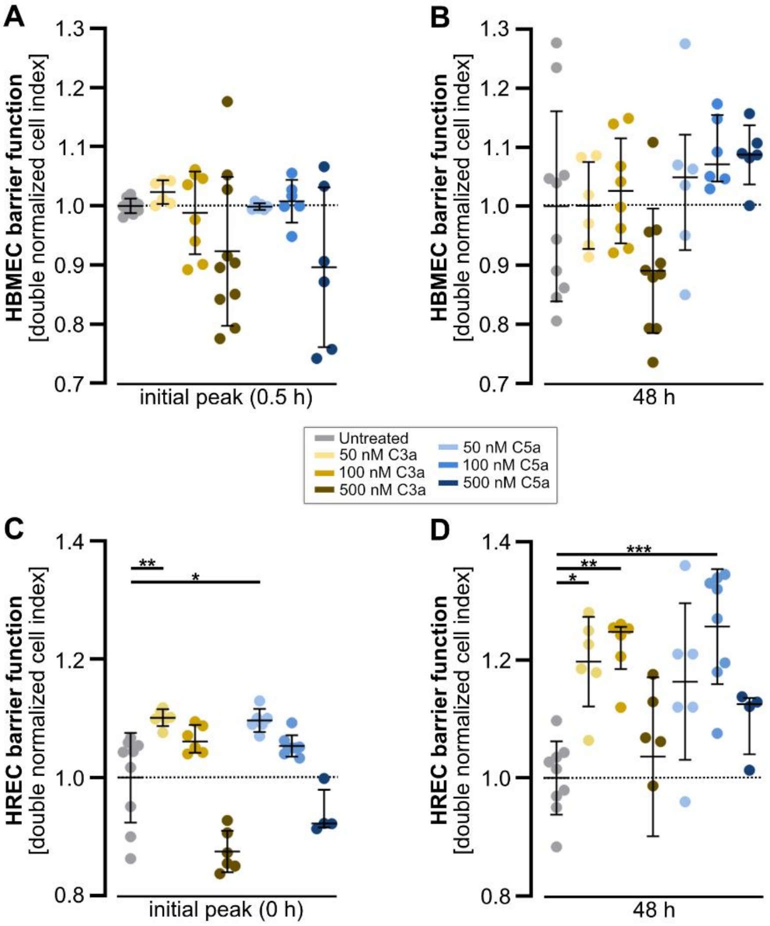

Supplement Figure S1 **Low-dose stimulation of HREC with anaphylatoxins C3a and C5a increase the dnCI. (A)** DnCI in HBMEC remained unaltered after exposure to 50 nM, 100 nM and 500 nM C3a and C5a for 0.5 hours. **(B)** After 48 hours of exposure to anaphylatoxins, there was no change in dnCI between treatment groups. (C) Immediately after treatment initiation, 50 nM C3a and 50 nM C5a significantly increased the dnCI in HREC when compared with the untreated control. (D) 48 hours after exposure to anaphylatoxins, the dnCI was significantly increased in 50 nM C3a, 100 nM C3a and 100 nM C5a treated HREC when compared with the untreated control. **(A, B)** RTCA graph represents data from *n* = 10 for untreated and 500 nM C3a, *n* = 6 for 50 nM C3a and 50, 100 and 500 nM C5a and *n* = 8 for 100 nM C3a treated cells, respectively. **(C, D)** RTCA graph represent data from *n* = 9 for

untreated, $n = 6$ for 50 nM C5a and 50, 100 and 500 nM C3a, $n = 8$ for 100 nM C5a and $n = 4$ for 500 nM C5a, respectively. **(A, B, C, D)** Two and 24 hour time points were analysed as separate datasets. Kruskal-Wallis Test with Dunn's multiple comparisons test was used for datasets including non-parametric data. One-way ANOVA with Dunnett's T3 post hoc test for parametric data sets. *p <.05, **p <.01, ***p <.001.

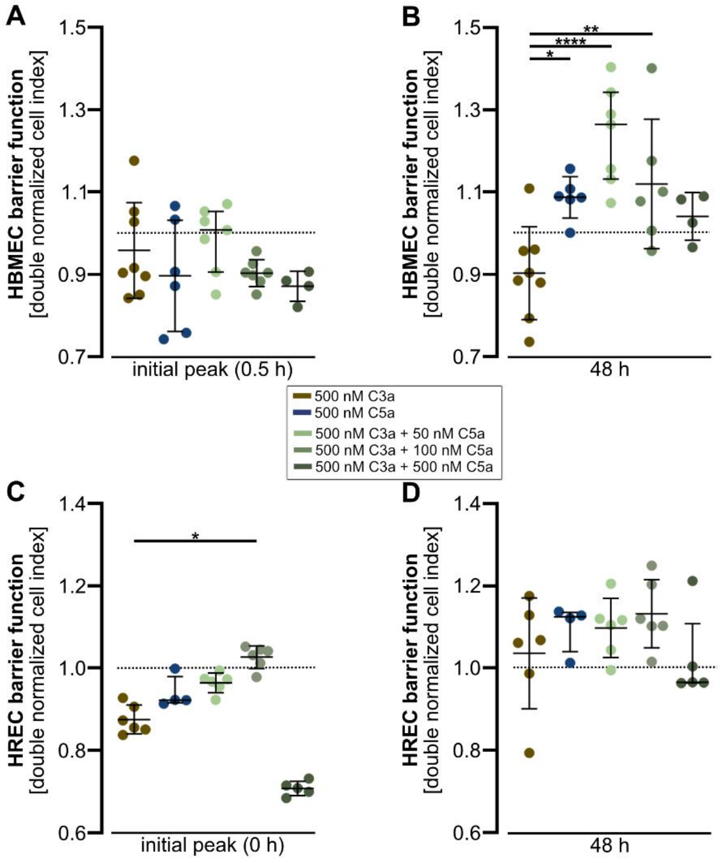

Supplement Figure S2 **C5a attenuates the C3a-mediated disruptive effect on the endothelial monolayer after 48 hours of exposure in HBMEC. (A)** 500 nM C3a + 50 nM C5a, 500 nM C3a + 100 nM C5a and 500 nM C3a + 500 nM C5a altered the dnCI in HBMEC 0.5 hours after treatment initiation when compared to 500 nM C3a treated HBMEC. **(B)** After 48 hours exposure to 500 nM C3a + 50 nM C5a and 500 nM C3a + 100 nM C5a significantly attenuated of the dnCI compared to 500 nM C3a treatment in HBMEC. **(C)** Immediately after treatment initiation, 500 nM C3a + 100 nM C5a increased the dnCI in HREC compared to 500 nM C3a treated cells. **(D)** The dnCI remained unaltered in all treatment groups after 48 hours of anaphylatoxin treatment in HREC when compared with 500 nM C3a treated cells. **(A, B)** RTCA graph represents the mean of data from $n = 9$ for 500 nM C3a, $n = 6$ for 500 nM C5a and $n = 7$ for 500 nM C3a + 50 nM C5a and 500 nM C3a + 100 nM C5a and $n = 4$ for 500 nM C3a + 500 nM C5a treated cells, respectively. **(C, D)** RTCA graph represents the mean of data from $n =$ 6 for 500 nM C3a, 500 nM C3a + 50 nM C5a and 500 nM C3a + 100 nM C5a and $n = 4$ for 500 nM C5a and $n = 5$ for 500 nM C3a + 500 nM C5a. **(A, B, C, D)** Two and 24 hour time points were analysed as separate datasets. Kruskal-Wallis Test with Dunn's multiple comparisons test was used for datasets including non-parametric data. One-way ANOVA with Dunnett's T3 post hoc test was used for parametric data sets. *p <.05, **p <.01, ****p <.0001.

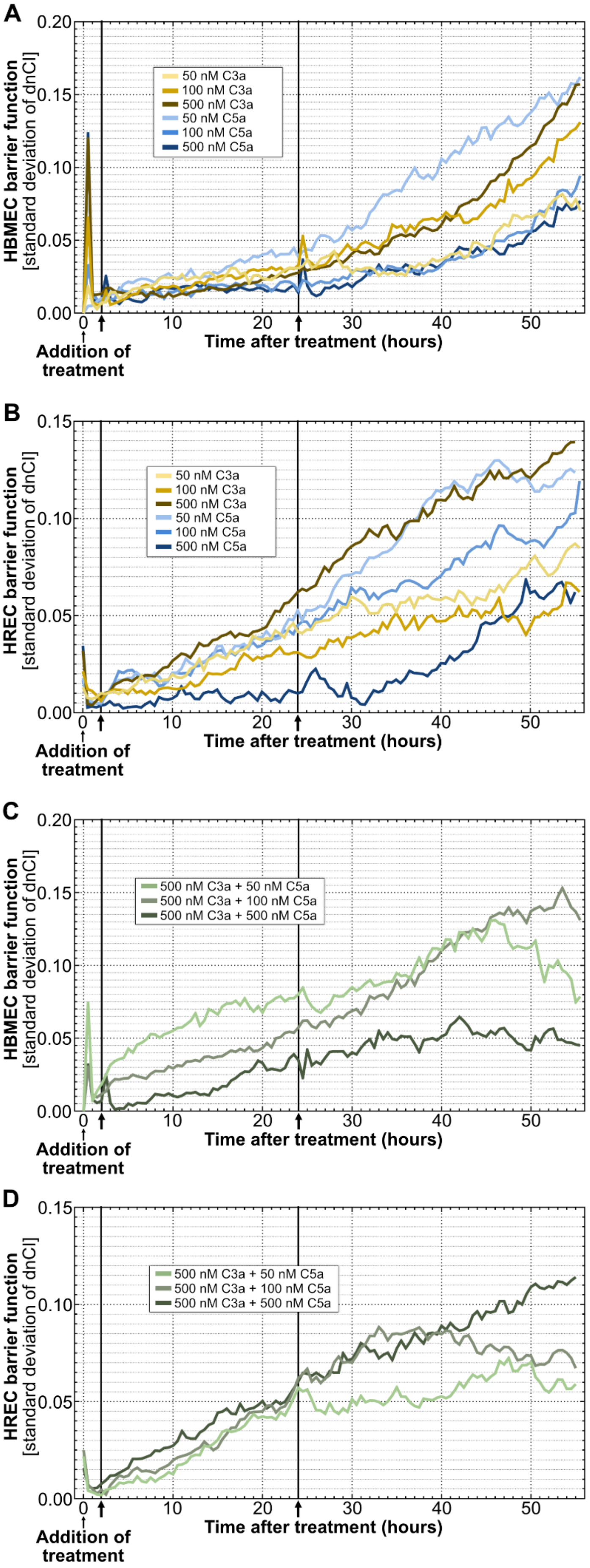
A
HBMEC barrier function
[standard deviation of dnCI]
0.20
0.15
0.10
0.05
0.00
50 nM C3a
100 nM C3a
500 nM C3a
50 nM C5a
100 nM C5a
500 nM C5a
0
10
20
30
40
50
Time after treatment (hours)
Addition of treatment
B
HREC barrier function
[standard deviation of dnCI]
0.15
0.10
0.05
0.00
50 nM C3a
100 nM C3a
500 nM C3a
50 nM C5a
100 nM C5a
500 nM C5a
0
10
20
30
40
50
Time after treatment (hours)
Addition of treatment
C
HBMEC barrier function
[standard deviation of dnCI]
0.20
0.15
0.10
0.05
0.00
500 nM C3a + 50 nM C5a
500 nM C3a + 100 nM C5a
500 nM C3a + 500 nM C5a
0
10
20
30
40
50
Time after treatment (hours)
Addition of treatment
D
HREC barrier function
[standard deviation of dnCI]
0.15
0.10
0.05
0.00
500 nM C3a + 50 nM C5a
500 nM C3a + 100 nM C5a
500 nM C3a + 500 nM C5a
0
10
20
30
40
50
Time after treatment (hours)
Addition of treatment

Supplement Figure S3 **The standard deviation of dnCI positively correlates with cultivation time across all treatment groups in the RTCA. (A)** The dnCI standard deviation peaked during the initial search in dnCI and increased over time in all HBMEC treatment groups, particularly after the 24 hour analysis time point. **(B)** DnCI increased in all HREC treatment groups, with 500 nM C5a maintaining a very low standard deviation until 40 hours after treatment. **(C)** The combination treatment of C3a and C5a showed an increasing standard deviation in dnCI over the time of HBMEC cultivation. **(D)** In HREC, the standard deviation among all combination treatments was similar until the 24 hours analysis timepoint, after which it remained stable in 500 nM C3a + 50 nM C5a treated cells. **(A)** RTCA graph represents data from *n* = 10 for untreated and 500 nM C3a, *n* = 6 for 50 nM C3a and 50, 100 and 500 nM C5a and *n* = 8 for 100 nM C3a treated cells, respectively. **(B)** The RTCA graph represent data from *n* = 9 for untreated, *n* = 6 for 50 nM C5a and 50, 100 and 500 nM C3a, *n* = 8 for 100 nM C5a and *n* = 4 for 500 nM C5a, respectively. **(C)** The RTCA graph represents the mean of data from *n* = 9 for 500 nM C3a, *n* = 6 for 500 nM C5a and *n* = 7 for 500 nM C3a + 50 nM C5a and 500 nM C3a + 100 nM C5a and *n* = 4 for 500 nM C3a + 500 nM C5a treated cells, respectively. **(D)** The RTCA graph represents the mean of data from *n* = 6 for 500 nM C3a, 500 nM C3a + 50 nM C5a and 500 nM C3a + 100 nM C5a and *n* = 4 for 500 nM C5a and *n* = 5 for 500 nM C3a + 500 nM C5a.

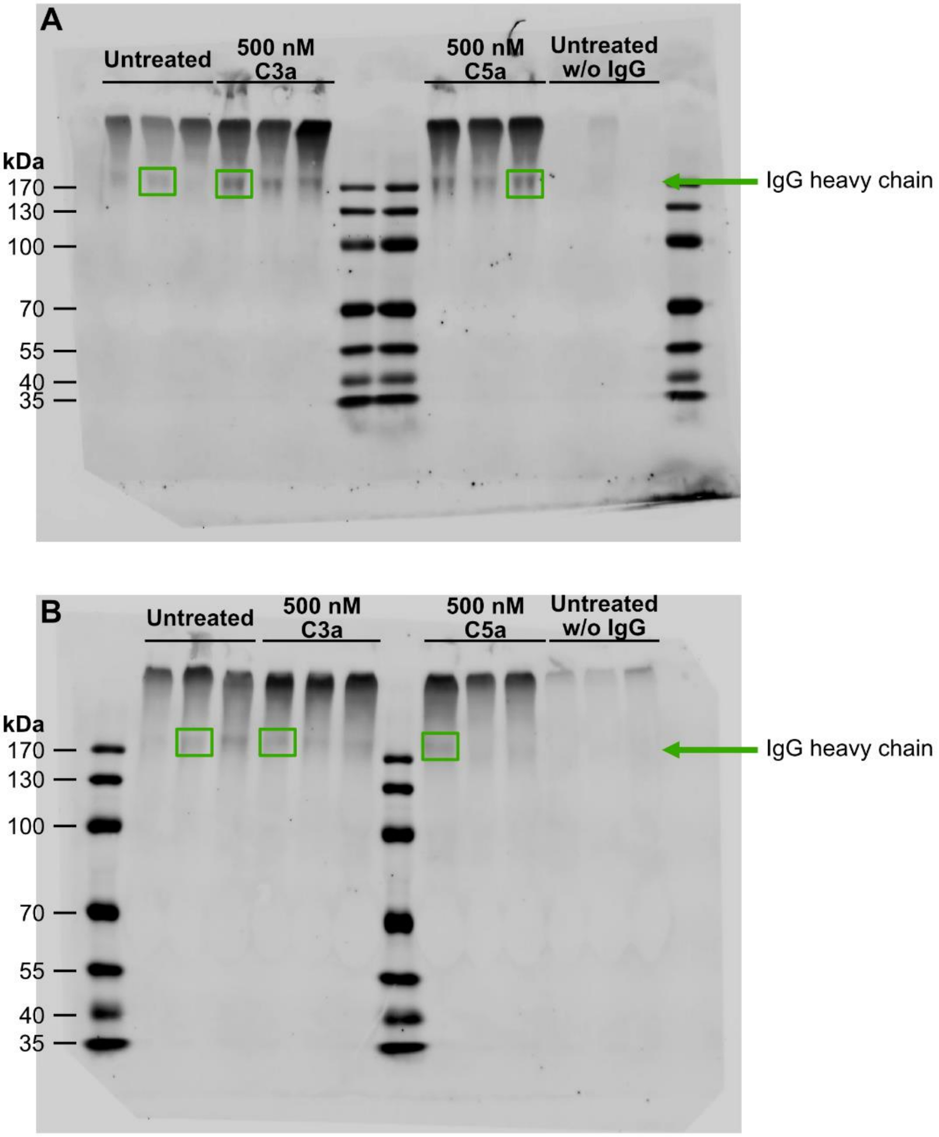

Supplement Figure S4 **Uncropped Western blots for (A) HBMEC and (B) HREC in Figure 8B.**

Supplement Table S1 **In-silico validation of in-house designed primers.**

General information		*in silico primer validation*								
		UCSC in silico PCR					NCBI Primer blast			Primer Design
**Transcript**	**Primer sequence**	**Product(s)**	**Ensembl ID**	**no. of amplicons**	**amplicon size**	**Target sequence (Top Hit)**	**NCBI blast (Top Hit)**	**amplicon size**	**mismatch**	**Category**
CDH5	GGACCGAGAGAAGCAGGCCA<br>TGTGTACTTGGTCTGGGTGAAGA	CDH5	ENST00000341529.8	3	148	GGACCGAGAGAAGCAGGCCAggt at gagat cgt ggt ggaagcgcg agat gcccagggcctccggg gggactcgggcacggccaccg tgctggtcactctgcaagacat caatgacaacttcccctTCTT CACCCAGACCAAGTACACA	NM_001795.5 Homo sapiens cadherin 5 (CDH5)	148	--	Ex5 Ex6 spanning
C3	AAGAACCGCTGGGAGGACCC<br>ATTGAGCCAACGCACGACGG	C3	ENST00000695652.1	8	123	AAGAACCGCTGGGAGGACCCtggtaagcagctctacaacgtggaggccacatcctatgccctcttggccctactgcagctaaaagactttgactttgtgcctcCCGTCGTGCGTTGGCTCAAT	C3	123	--	Within exon 29
C3AR1	TTCCGAATGCAAAGGGGCCG<br>ACCACGGCCACTCGAAAGGT	C3AR1	ENST00000307637.5	1	62	TTCCGAATGCAAAGGGGCCGcttcgccaagtctcaggcaaaACCTTTCGAGTGGCCGTGGT	NM_001326477.2 Complement C3a receptor 1 (C3AR1), transcript variant 3 -1	62	--	Within exon 2
GAPDH	CCCCACCACACTGAATCTCC<br>GGTACTTTATTGATGGTACATGACAAG	GAPDH	ENST00000229239.10	4	105	CCCCACCACACTGAATCTCCcctcctcacagttgccatgtagaccccttgaagaggggaggggcctagggagccgcacCTTGTCATGTACCATCAATAAAGTACC	NM_001357943.2 GAPDH, transcript variant 7	105	--	Ex1 Ex3 spanning Ex2 Ex4 spanning

Supplement Table S2 **Wet lab validation of in-house designed primers.**

Primer set	PCR product size	Forward Primer	Sequence (bold nucleotides align with NCBI Top Hit	Reverse Primer	NCBI Blast (Top Hit)	Sequence ID
CDH5	161	GGACCGAGAGAAGCAGGCCA	GGATTTGTAGTAGCGCGAGATGCCCAGGGCCTCCGGGGGGACTCGGGCACGGCCACCGTGCTGGTCACTCTGCAAGACATCAATGACAACTTCCCCTTCTTCACCCAGACCAAGTACACAAACTC		cadherin 5 (CDH5)	NM_001795.5
			GGATTGTATTGATGTCTTGCAGAGTGACCAGCACGGTGGCCGTGCCCGAGTCCCCCCGGAGGCCCTGGGCATCTCGCGCTTCCACCACGATCTCATACCTGGCCTGCTTCTCTCGGTCCAAGCGTACCCT	TGTGTACTTGGTCTGGGTGAAGA	cadherin 5 (CDH5)	NM_001795.5
GAPDH	105	CCCCACCACACTGAATCTCC	AGGGAAGGGGGCCTAGGGAGCCGCACCTTGTCATGTACCATCAATAAAGTACCAATTCG		glyceraldehyde-3-phosphate dehydrogenase (GAPDH)	NM_001289745.3
			CTCGTCTCAAGGGGGTCTACATGGCAACTGTGAGGAGGGGAGATTCAGTGTGGTGGGGAAT	GGTACTTTATTGATGGTACATGACAAG	glyceraldehyde-3-phosphate dehydrogenase (GAPDH)	NM_001289745.3
